# *N*,*N*,*N*′,*N*′-Tetra­kis(pyridin-4-yl)methane­diamine monohydrate

**DOI:** 10.1107/S1600536812019010

**Published:** 2012-05-02

**Authors:** Jong Won Shin, Kil Sik Min

**Affiliations:** a Department of Chemistry, Kyungpook National University, Daegu 702-701, Republic of Korea; b Department of Chemistry Education, Kyungpook National University, Daegu 702-701, Republic of Korea

## Abstract

In the title compound, C_21_H_18_N_6_·H_2_O, two 4,4′-dipyridyl­amine groups are linked by a methyl­ene C atom, which sits on a twofold axis. The lattice water mol­ecule is located slightly off a twofold axis, and is therefore disordered over two positions. In the crystal, the organic mol­ecules and the water mol­ecule are linked by O—H⋯N hydrogen bonds. The organic mol­ecules exhibit extensive offset face-to-face π–π inter­actions to symmetry equivalents [centroid–centroid distances = 3.725 (3) and 4.059 (3) Å].

## Related literature

For metal-organic frameworks including 4,4′-dipyridyl­amine, see: Braverman & LaDuca (2007 ▶); Shyu *et al.* (2009 ▶). For the catalysis of multidimensional metal-organic frameworks, see: Welbes & Borovik (2005 ▶). For self-assembled metal-organic networks and their luminescent properties, see: Shin *et al.* (2012 ▶); Zeng *et al.* (2010 ▶).
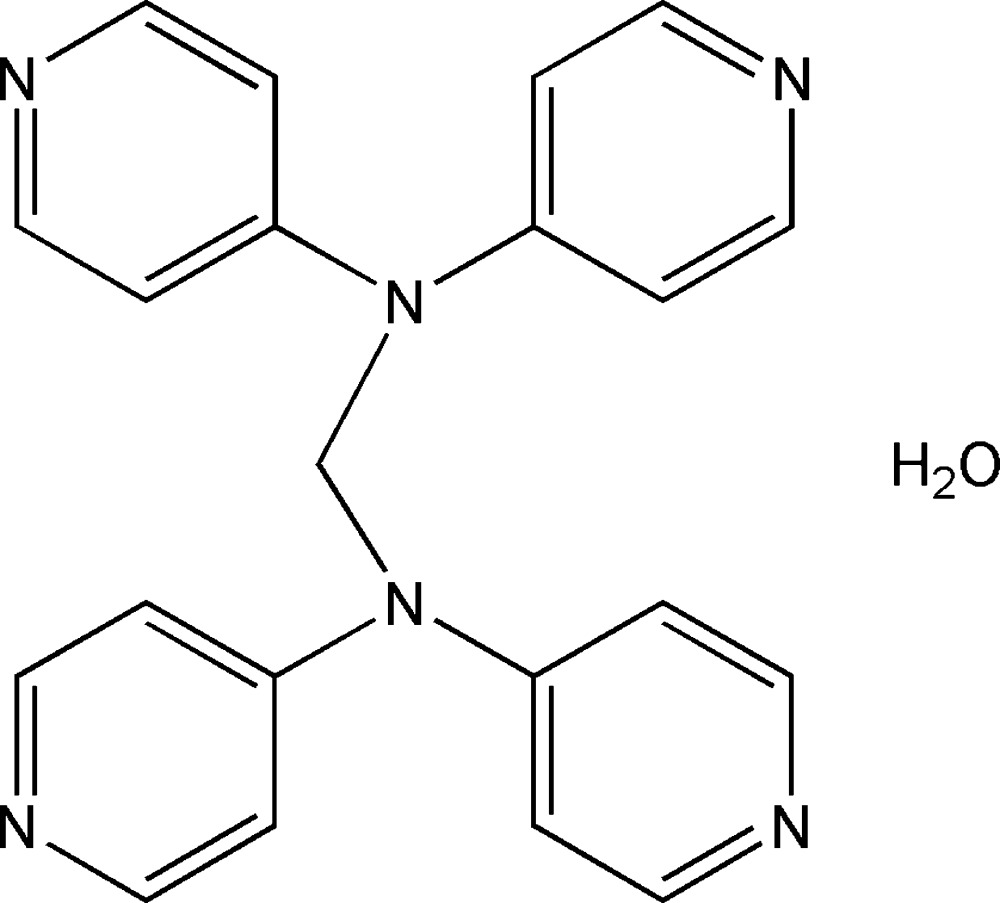


## Experimental

### 

#### Crystal data

C_21_H_18_N_6_·H_2_O
*M* *_r_* = 372.42
Monoclinic, 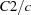

*a* = 13.9048 (11) Å
*b* = 13.7637 (11) Å
*c* = 10.0569 (8) Å
β = 109.142 (2)°
*V* = 1818.3 (3) Å^3^
*Z* = 4
Mo *K*α radiation
μ = 0.09 mm^−1^
*T* = 200 K
0.34 × 0.26 × 0.25 mm

#### Data collection

Siemens SMART CCD diffractometer
Absorption correction: multi-scan (*SADABS*; Sheldrick, 1996 ▶) *T*_min_ = 0.973, *T*_max_ = 0.978
6540 measured reflections
2241 independent reflections
1313 reflections with *I* > 2σ(*I*)
*R*_int_ = 0.033

#### Refinement

*R*[*F* ^2^ > 2σ(*F* ^2^)] = 0.051
*wR*(*F* ^2^) = 0.163
*S* = 1.09
2241 reflections
130 parameters
H-atom parameters constrained
Δρ_max_ = 0.23 e Å^−3^
Δρ_min_ = −0.22 e Å^−3^

### 

Data collection: *SMART* (Siemens, 1996 ▶); cell refinement: *SAINT* (Siemens, 1996 ▶); data reduction: *SAINT*; program(s) used to solve structure: *SHELXS97* (Sheldrick, 2008 ▶); program(s) used to refine structure: *SHELXL97* (Sheldrick, 2008 ▶); molecular graphics: *ORTEP-3 for Windows* (Farrugia, 1997 ▶); software used to prepare material for publication: *SHELXL97*.

## Supplementary Material

Crystal structure: contains datablock(s) global, I. DOI: 10.1107/S1600536812019010/pk2409sup1.cif

Structure factors: contains datablock(s) I. DOI: 10.1107/S1600536812019010/pk2409Isup2.hkl

Supplementary material file. DOI: 10.1107/S1600536812019010/pk2409Isup3.cml

Additional supplementary materials:  crystallographic information; 3D view; checkCIF report

## Figures and Tables

**Table 1:** Hydrogen-bond geometry (Å, °)

*D*—H⋯*A*	*D*—H	H⋯*A*	*D*⋯*A*	*D*—H⋯*A*
O1—H1*W*⋯N2^i^	1.02	1.88	2.869 (2)	161

Symmetry code: (i) 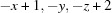.

**Fig. 1.**
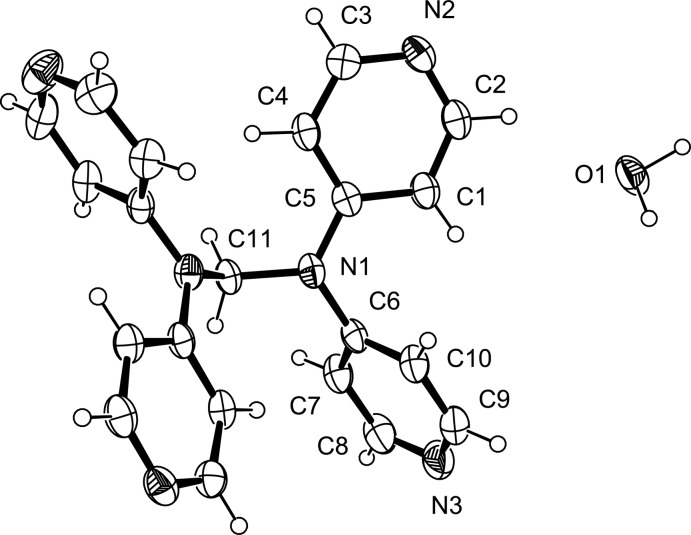
An ellipsoid plot (40% probability) of the title compound. The unlabelled half of the molecule is related by a crystallographic 2-fold axis. The water molecule is disordered about a 2-fold axis (for clarity, only one component is shown).

**Fig. 2.**
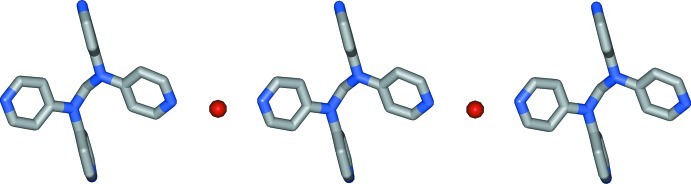
A view of the title compound showing a one-dimensional chain formed by O—H···N hydrogen bonding interactions. The chain extends along the (101) direction.

**Fig. 3.**
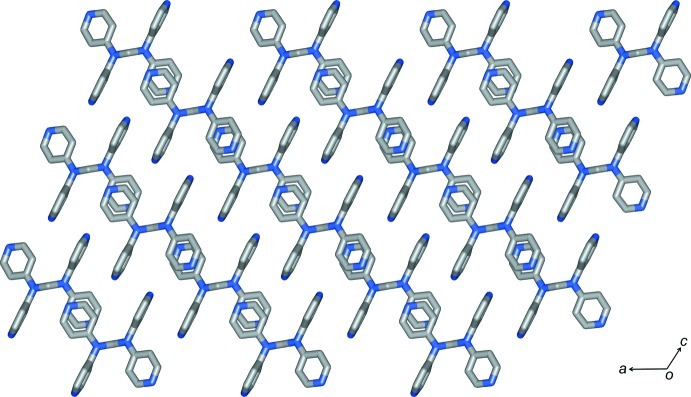
A view of the title compound showing offset face-to-face π-π interactions.

## supplementary crystallographic information

### Comment

Polypyridyl ligands have attracted considerable attention in materials science because they can be used for the construction of multidimensional metal-organic frameworks. These have potential applications in catalysis and as luminescent materials (Shin *et al.*, 2012; Welbes & Borovik, 2005; Zeng *et al.*, 2010). For example, as a building block, bis(4-pyridyl)amine (bpa) has been extensively used for self-assembly of multidimensional coordination polymers, because the ligand has significant functionalities, *e.g.* hydrogen bonding capability (Braverman & LaDuca, 2007; Shyu, *et al.*, 2009). Thus, we have made a new ligand, *N,N,N',N'*-tetra-4-pyridyl-methylenediamine (TPMD), which can be used as a building unit for self-assembly of potential luminescent materials and catalysts. Here, we report the synthesis and crystal structure of *N,N,N',N'*-tetra-4-pyridyl-methylenediamine monohydrate.

The title compound in its crystalline state is centrosymmetric (Fig. 1). The dihedral angle between neighboring pyridyl rings is 63.74 (7)°, and the angle of N1—C11—N1(-*x*, *y*, 1.5 - *z*) is 114.5 (2)°. The water molecule appears to be slightly off a 2-fold axis, and was refined using a disordered model, which gave a lower *R* value and a flatter difference map compared to a non-disordered model. The crystal packing is stabilized by strong intermolecular O—H···N hydrogen bonds (Table 1) that connect pairs of organic molecules by water molecules into chains along the (101) direction (Fig. 2). The crystal is also stabilized by intermolecular offset face-to-face π-π interactions [centroid-centroid distances = 3.725 (3) Å (-*x* + 1/2, -*y* + 1/2, 2 - *z*) and 4.059 (3) Å (-*x* + 1/2, -*y* + 1/2, 1 - *z*)] (Fig. 3).

### Experimental

The title compound was prepared as follows. NaH (0.561 g, 0.0234 mol) was added carefully to a DMF solution (50 ml) of 4,4'-dipyridylamine (2.00 g, 0.0117 mol) and stirred for 2 days at room temperature. To the mixture was added dropwise dichloromethane (20 ml) and the mixture solution was again stirred for 2 days, which resulted in a dark red solution. Then the mixture was quenched with H_2_O (50 ml), and the organic layer was extracted with CHCl_3_ (3 times, 100 ml). The extract was washed with NaCl solution to purify and then dried with Na_2_SO_4_. After removing the organic solvent, a pale yellow oil was obtained, from which colorless crystals formed in 1 day. The crystals were filtered and washed with *n*-hexane and acetonitrile. Yield: 0.86 g (42%). Anal. Calcd. for C_21_H_20_N_6_O: C, 67.73; H, 5.41; N, 22.57. Found: C, 67.63; H, 5.23; N, 22.51. ^1^H NMR (400 MHz, DMSO-d^6^, 300 K): *δ* = 8.35 (dd, J = 1.52, 1.52 Hz, 8 H), 6.91 (dd, J = 1.56, 1.60 Hz, 8 H), 5.95 (s, 2H). GC—MS: m/*z* = 354.1 (*M*^+^). IR (KBr, cm^-1^): 3425, 3050, 3024, 1601, 1581, 1497, 1207, 1068, 850, 602.

### Refinement

The H atom of O1 was located in a difference Fourier map and refined isotropically. The remaining H atoms were placed in geometrically idealized positions and constrained to ride on their parent atoms, with C—H distances of 0.95 (ring H atoms) Å and 0.99 (open chain H atoms) Å, and with *U*_iso_(H) values of 1.2 times the equivalent anisotropic displacement parameters of the parent C atoms.

### Crystal data

**Table:**

C_21_H_18_N_6_·H_2_O	*F*(000) = 784
*M**_r_* = 372.42	*D*_x_ = 1.360 Mg m^−^^3^
Monoclinic, *C*2/*c*	Mo *K*α radiation, λ = 0.71073 Å
Hall symbol: -C 2yc	Cell parameters from 2155 reflections
*a* = 13.9048 (11) Å	θ = 2.7–28.2°
*b* = 13.7637 (11) Å	µ = 0.09 mm^−^^1^
*c* = 10.0569 (8) Å	*T* = 200 K
β = 109.142 (2)°	Block, colorless
*V* = 1818.3 (3) Å^3^	0.34 × 0.26 × 0.25 mm
*Z* = 4	

### Data collection

**Table:**

Siemens SMART CCD diffractometer	2241 independent reflections
Radiation source: fine-focus sealed tube	1313 reflections with *I* > 2σ(*I*)
Graphite monochromator	*R*_int_ = 0.033
φ and ω scans	θ_max_ = 28.3°, θ_min_ = 2.1°
Absorption correction: multi-scan (*SADABS*; Sheldrick, 1996)	*h* = −14→18
*T*_min_ = 0.973, *T*_max_ = 0.978	*k* = −18→16
6540 measured reflections	*l* = −13→12

### Refinement

**Table:**

Refinement on *F*^2^	Primary atom site location: structure-invariant direct methods
Least-squares matrix: full	Secondary atom site location: difference Fourier map
*R*[*F*^2^ > 2σ(*F*^2^)] = 0.051	Hydrogen site location: inferred from neighbouring sites
*wR*(*F*^2^) = 0.163	H-atom parameters constrained
*S* = 1.09	*w* = 1/[σ^2^(*F*_o_^2^) + (0.0744*P*)^2^ + 0.1675*P*] where *P* = (*F*_o_^2^ + 2*F*_c_^2^)/3
2241 reflections	(Δ/σ)_max_ < 0.001
130 parameters	Δρ_max_ = 0.23 e Å^−^^3^
0 restraints	Δρ_min_ = −0.22 e Å^−^^3^

### Special details

**Table:**

Geometry. All e.s.d.'s (except the e.s.d. in the dihedral angle between two l.s. planes) are estimated using the full covariance matrix. The cell e.s.d.'s are taken into account individually in the estimation of e.s.d.'s in distances, angles and torsion angles; correlations between e.s.d.'s in cell parameters are only used when they are defined by crystal symmetry. An approximate (isotropic) treatment of cell e.s.d.'s is used for estimating e.s.d.'s involving l.s. planes.
Refinement. Refinement of *F*^2^ against ALL reflections. The weighted *R*-factor *wR* and goodness of fit *S* are based on *F*^2^, conventional *R*-factors *R* are based on *F*, with *F* set to zero for negative *F*^2^. The threshold expression of *F*^2^ > σ(*F*^2^) is used only for calculating *R*-factors(gt) *etc*. and is not relevant to the choice of reflections for refinement. *R*-factors based on *F*^2^ are statistically about twice as large as those based on *F*, and *R*- factors based on ALL data will be even larger.

### Fractional atomic coordinates and isotropic or equivalent isotropic displacement parameters (Å^2^)

**Table:**

	*x*	*y*	*z*	*U*_iso_*/*U*_eq_	Occ. (<1)
N1	0.08225 (11)	0.18977 (10)	0.72834 (16)	0.0378 (4)	
N2	0.32207 (12)	0.08225 (11)	1.07690 (17)	0.0456 (4)	
N3	0.12205 (15)	0.19990 (14)	0.3297 (2)	0.0627 (6)	
C1	0.24926 (14)	0.11276 (13)	0.8290 (2)	0.0415 (5)	
H1	0.2569	0.1085	0.7387	0.050*	
C2	0.32458 (14)	0.07990 (13)	0.9453 (2)	0.0455 (5)	
H2	0.3836	0.0532	0.9315	0.055*	
C3	0.23734 (15)	0.12101 (13)	1.0907 (2)	0.0444 (5)	
H3	0.2327	0.1249	1.1827	0.053*	
C4	0.15609 (14)	0.15572 (12)	0.9798 (2)	0.0388 (5)	
H4	0.0977	0.1813	0.9966	0.047*	
C5	0.16063 (13)	0.15281 (11)	0.84383 (19)	0.0356 (4)	
C6	0.09476 (13)	0.19319 (12)	0.5933 (2)	0.0373 (4)	
C7	0.09792 (15)	0.28090 (13)	0.5269 (2)	0.0461 (5)	
H7	0.0918	0.3406	0.5708	0.055*	
C8	0.11000 (16)	0.28004 (16)	0.3965 (2)	0.0569 (6)	
H8	0.1097	0.3407	0.3512	0.068*	
C9	0.11687 (16)	0.11622 (16)	0.3940 (2)	0.0555 (6)	
H9	0.1238	0.0577	0.3480	0.067*	
C10	0.10220 (14)	0.10896 (14)	0.5221 (2)	0.0452 (5)	
H10	0.0972	0.0471	0.5613	0.054*	
C11	0.0000	0.24732 (13)	0.7500	0.0355 (6)	
H11A	0.0293	0.2898	0.8329	0.043*	0.50
H11B	−0.0293	0.2898	0.6671	0.043*	0.50
O1	0.4878 (6)	0.01031 (13)	0.7761 (8)	0.0617 (12)	0.50
H1W	0.5532	−0.0300	0.8088	0.130 (11)*	

### Atomic displacement parameters (Å^2^)

**Table:**

	*U*^11^	*U*^22^	*U*^33^	*U*^12^	*U*^13^	*U*^23^
N1	0.0315 (8)	0.0354 (8)	0.0500 (9)	0.0046 (6)	0.0180 (7)	0.0039 (6)
N2	0.0331 (9)	0.0417 (9)	0.0577 (11)	0.0006 (7)	0.0089 (8)	0.0033 (7)
N3	0.0592 (13)	0.0774 (13)	0.0613 (12)	0.0230 (10)	0.0331 (10)	0.0175 (10)
C1	0.0353 (11)	0.0379 (10)	0.0547 (12)	0.0007 (8)	0.0193 (9)	0.0021 (8)
C2	0.0335 (11)	0.0403 (10)	0.0650 (14)	0.0014 (8)	0.0190 (9)	−0.0005 (9)
C3	0.0454 (12)	0.0394 (10)	0.0498 (12)	−0.0056 (9)	0.0175 (9)	0.0005 (8)
C4	0.0315 (10)	0.0314 (9)	0.0577 (12)	−0.0013 (7)	0.0203 (9)	0.0024 (8)
C5	0.0319 (10)	0.0260 (8)	0.0497 (11)	−0.0037 (7)	0.0146 (8)	0.0022 (7)
C6	0.0262 (9)	0.0382 (10)	0.0498 (11)	0.0035 (7)	0.0158 (8)	0.0066 (8)
C7	0.0407 (12)	0.0382 (10)	0.0663 (14)	0.0027 (8)	0.0270 (10)	0.0090 (9)
C8	0.0490 (14)	0.0604 (14)	0.0709 (15)	0.0124 (10)	0.0326 (11)	0.0269 (11)
C9	0.0514 (14)	0.0595 (13)	0.0595 (14)	0.0176 (10)	0.0235 (11)	0.0006 (10)
C10	0.0433 (12)	0.0387 (10)	0.0572 (12)	0.0078 (8)	0.0214 (10)	0.0033 (8)
C11	0.0285 (13)	0.0291 (12)	0.0509 (15)	0.000	0.0158 (11)	0.000
O1	0.052 (2)	0.0452 (13)	0.072 (2)	0.0162 (17)	−0.0008 (17)	−0.0107 (16)

### Geometric parameters (Å, º)

**Table:**

N1—C5	1.402 (2)	C4—H4	0.9500
N1—C6	1.425 (2)	C6—C10	1.384 (3)
N1—C11	1.4649 (17)	C6—C7	1.387 (2)
N2—C2	1.335 (2)	C7—C8	1.376 (3)
N2—C3	1.341 (2)	C7—H7	0.9500
N3—C8	1.330 (3)	C8—H8	0.9500
N3—C9	1.335 (3)	C9—C10	1.373 (3)
C1—C2	1.366 (3)	C9—H9	0.9500
C1—C5	1.402 (2)	C10—H10	0.9500
C1—H1	0.9500	C11—N1^i^	1.4649 (17)
C2—H2	0.9500	C11—H11A	0.9900
C3—C4	1.387 (3)	C11—H11B	0.9900
C3—H3	0.9500	O1—O1^ii^	0.7127
C4—C5	1.390 (2)	O1—H1W	1.0230
			
C5—N1—C6	119.78 (14)	C10—C6—N1	121.22 (15)
C5—N1—C11	120.38 (13)	C7—C6—N1	121.38 (16)
C6—N1—C11	117.96 (12)	C8—C7—C6	118.99 (18)
C2—N2—C3	114.92 (16)	C8—C7—H7	120.5
C8—N3—C9	115.77 (19)	C6—C7—H7	120.5
C2—C1—C5	119.54 (18)	N3—C8—C7	124.30 (18)
C2—C1—H1	120.2	N3—C8—H8	117.8
C5—C1—H1	120.2	C7—C8—H8	117.8
N2—C2—C1	125.35 (19)	N3—C9—C10	124.49 (19)
N2—C2—H2	117.3	N3—C9—H9	117.8
C1—C2—H2	117.3	C10—C9—H9	117.8
N2—C3—C4	124.43 (18)	C9—C10—C6	118.94 (17)
N2—C3—H3	117.8	C9—C10—H10	120.5
C4—C3—H3	117.8	C6—C10—H10	120.5
C3—C4—C5	119.59 (17)	N1^i^—C11—N1	114.54 (16)
C3—C4—H4	120.2	N1^i^—C11—H11A	108.6
C5—C4—H4	120.2	N1—C11—H11A	108.6
C4—C5—C1	116.16 (16)	N1^i^—C11—H11B	108.6
C4—C5—N1	122.06 (16)	N1—C11—H11B	108.6
C1—C5—N1	121.76 (17)	H11A—C11—H11B	107.6
C10—C6—C7	117.39 (18)	O1^ii^—O1—H1W	69.5
			
C3—N2—C2—C1	−0.3 (3)	C11—N1—C6—C10	−129.57 (17)
C5—C1—C2—N2	0.0 (3)	C5—N1—C6—C7	−115.35 (19)
C2—N2—C3—C4	0.8 (3)	C11—N1—C6—C7	49.1 (2)
N2—C3—C4—C5	−1.1 (3)	C10—C6—C7—C8	−1.2 (3)
C3—C4—C5—C1	0.7 (2)	N1—C6—C7—C8	−179.92 (17)
C3—C4—C5—N1	−177.87 (15)	C9—N3—C8—C7	3.2 (3)
C2—C1—C5—C4	−0.2 (2)	C6—C7—C8—N3	−2.1 (3)
C2—C1—C5—N1	178.37 (16)	C8—N3—C9—C10	−1.2 (3)
C6—N1—C5—C4	174.20 (15)	N3—C9—C10—C6	−1.8 (3)
C11—N1—C5—C4	10.1 (2)	C7—C6—C10—C9	2.9 (3)
C6—N1—C5—C1	−4.3 (2)	N1—C6—C10—C9	−178.31 (17)
C11—N1—C5—C1	−168.41 (15)	C5—N1—C11—N1^i^	−82.93 (13)
C5—N1—C6—C10	65.9 (2)	C6—N1—C11—N1^i^	112.69 (14)

Symmetry codes: (i) −*x*, *y*, −*z*+3/2; (ii) −*x*+1, *y*, −*z*+3/2.

### Hydrogen-bond geometry (Å, º)

**Table:**

*D*—H···*A*	*D*—H	H···*A*	*D*···*A*	*D*—H···*A*
O1—H1*W*···N2^iii^	1.02	1.88	2.869 (2)	161

Symmetry code: (iii) −*x*+1, −*y*, −*z*+2.
